# Juno Observations of Jupiter's Magnetodisk Plasma: Implications for Equilibrium and Dynamics

**DOI:** 10.1029/2024JA032976

**Published:** 2024-11-23

**Authors:** Z.‐Y. Liu, M. Blanc, N. Andre, F. Bagenal, R. J. Wilson, F. Allegrini, M. Devinat, B. Mauk, J. E. P. Connerney, S. Bolton

**Affiliations:** ^1^ Institut de Recherche en Astrophysique et Planetologie (IRAP) CNES‐CNRS‐Universite Toulouse III Paul Sabatier Toulouse France; ^2^ Laboratoire d’Astrophysique de Marseille Aix‐Marseille Université CNRS Marseille France; ^3^ Institut Supérieur de l’Aéronautique et de l’Espace (ISAE‐SUPAERO) Université de Toulouse Toulouse France; ^4^ Laboratory for Atmospheric and Space Physics University of Colorado Boulder Boulder CO USA; ^5^ Southwest Research Institute San Antonio TX USA; ^6^ Department of Physics and Astronomy University of Texas at San Antonio San Antonio TX USA; ^7^ Johns Hopkins University Applied Physics Laboratory Laurel MD USA; ^8^ NASA‐Goddard Space Flight Center Greenbelt MD USA

**Keywords:** Jovian magnetodisk, magnetodisk, Jupiter's magnetosphere, JADE, JEDI, Jupiter plasma sheet

## Abstract

The Jovian magnetodisk plays an essential role in the dynamics of the Jupiter system by coupling its various components. Here, we investigate the Juno (JADE, JEDI, and MAG) observations of the magnetodisk within 20–80 Jupiter radii ($R_{J}$) in the 0–6 hr local time sector. JADE and JEDI data are combined to generate equatorial plane distributions of density, pressure, temperature, and anisotropy of electrons, protons, and heavy ions. Results show: (a) Heavy ions dominate both the number density and pressure. (b) The number density and pressure of all species decrease with radial distance. (c) The temperature increases for electrons and heavy ions and decreases for protons as radial distance increases. (d) On average, the parallel pressure exceeds the perpendicular pressure for all species. Based on these distributions, we explore the equilibrium and dynamics of the magnetodisk and show that: (a) Radial force balance is primarily achieved between the inward magnetic stress and the outward plasma anisotropy force. (b) An examination of the kappa parameters indicates that electrons, protons, and heavy ions primarily undergo adiabatic motion, magnetic moment diffusion, and stochastic motion, respectively. (c) A radial diffusion coefficient is derived from the radial profile of mass, providing an estimate of the timescale for radial transport from 20 to 80 $R_{J}$ of ∼ 7 hr (d) The total mass ($5.0\times 10^{7}$ kg) and thermal energy ($3.8\times 10^{37}$ eV) of the magnetodisk between 20 and 80 $R_{J}$ are obtained.

## Key Points

- Spatial distributions of the Jovian magnetodisk plasma parameters are derived from Juno/JADE+JEDI data during Juno's first 25 orbits
- Radial force balance and radial mass transport are discussed based on these distributions
- Our results provide constrains to modeling work and serve as a reference for future mission design

## 1 Introduction

**Figure 1:**
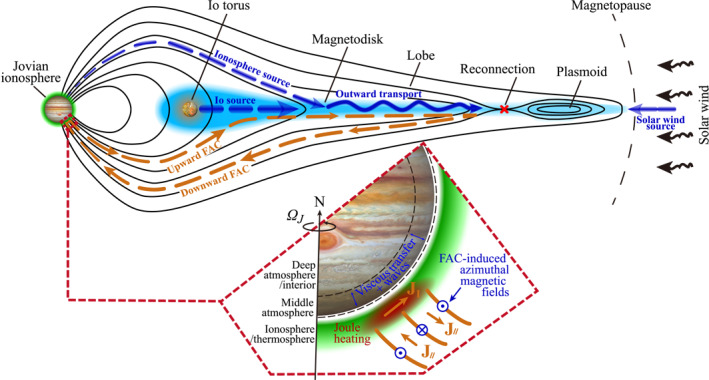
Schematic of the Jovian magnetodisk and its connection to other components of the Jupiter system. The black curves illustrate the magnetic field lines. The blue dashed arrows indicate the three primary mass sources of the magnetodisk: the Io torus (and possibly other moons), Jovian ionosphere, and the solar wind. The blue solid arrow indicates the outward transport of cold plasma (Inward injection of hot plasma is not shown here for figure clarity). The orange dashed curves illustrate the large‐scale electric currents connecting the magnetodisk and the ionosphere, as predicted by the corotation enforcement model. The inset illustrates how this current system is coupled and interacts with the atmosphere. For clarity, only the electric currents in the southern hemisphere and ionosphere mass source in the northern hemisphere are shown. However, they are supposed to also exist in the opposite hemisphere, although observations indicate that the detailed amounts may be different.

The Jovian magnetodisk (Figure 1, blue region) refers to a thin disc of plasma and electric current located near the equatorial plane beyond the orbit of the moon Io (e.g., Connerney et al., 1981; Khurana & Kivelson, 1989; Kivelson et al., 1978). It is believed to play an essential role in the dynamics of the Jupiter system, as it interacts with many of its components (Achilleos et al., 2015; Bagenal, 2007, and reference therein). First of all, the magnetodisk is coupled to the Io plasma torus, which is its dominant plasma source, and with which it exchanges mass, energy and momentum via radial transport likely driven by the interchange instability and larger scale transport processes (e.g., Y. Wang et al., 2022, 2023, and references therein). At the other end of the flow of cold plasma, it is coupled with the outer magnetosphere and magnetic tail, where the plasma is mainly lost from the system. The magnetodisk is also coupled with the upper atmosphere/ionosphere (Figure 1, green region) via field‐aligned currents (FACs; Figure 1, orange curves) (Al Saati et al., 2022; Khurana, 2001; Kotsiaros et al., 2019; Liu et al., 2023; Ray et al., 2014; Y. Wang et al., 2021). This current system is partially responsible for (or at least related to) the main aurora emissions and the heating of the upper atmosphere/ionosphere (Al Saati et al., 2022; Cowley & Bunce, 2001; Hill, 2001; Kamran et al., 2022; Kotsiaros et al., 2019; Y. Wang et al., 2021), and also provides a pathway of energy, momentum and angular moment transfer between the planet, upper atmosphere/ionosphere, magnetosphere and moons (e.g., Cowley & Bunce, 2001; Hill, 2001). The magnetodisk also displays specific interactions with each of the icy Galilean moons. On one hand, these interactions influence the space surrounding these moons and contribute to the shaping of their surfaces (e.g., Allegrini, Bagenal, et al., 2022, 2024; Khurana et al., 2007), as they regularly cross the magnetodisk, about twice per Jovian rotation, and expose their surfaces to the magnetodisk's particles (e.g., Bagenal et al., 2015; Liuzzo et al., 2022; Pelcener et al., 2024; Poppe et al., 2018; Santos et al., 2024). On the other hand, the magnetodisk gains mass from these moons, particularly from Europa and its torus (Bagenal & Delamere, 2011; Smyth & Marconi, 2006). However, these sources are less significant than Io. For instance, Smyth and Marconi (2006) estimated that the mass loading rate from Europa and its torus is about 22–27 kg/s, one or two orders of magnitude lower than that of the Io plasma torus. Finally, the magnetodisk hosts many microphysical processes, including reconnection between the magnetic fields of the northern and southern lobes (e.g., Russell et al., 1998), plasma instabilities (e.g., Saur et al., 2018), and turbulent cascade and dissipation (e.g., Saur et al., 2002). Hot plasma generated from these processes may then be transported inward and contribute to the dynamics of the inner magnetosphere, including the Io torus (Siscoe et al., 1981; Southwood & Kivelson, 1987; Tsuchiya et al., 2019) and even the radiation belts.

Determining the properties of magnetodisk plasma is an indispensable step toward a comprehensive description of magnetodisk physics. Since the 1970s, the magnetodisk has been visited by many spacecraft missions equipped with plasma instruments, establishing a basic picture. Now, we know the magnetodisk is mainly composed of electrons, protons, oxygen ions and sulfur ions (Kane et al., 1995; Kim et al., 2020; Krimigis et al., 1981; Liu et al., 2021; Mauk et al., 1996, 2004; J.‐Z. Wang et al., 2024; Wilson, 2024). The last two species, which primarily come from Io's escaping atmosphere (?Delamere et al., 2005), contribute a significant or even dominant fraction of the total mass and energy density. The first three moments, that is, density, momentum and pressure, of these species have been derived (e.g., Kane et al., 1995; Kim et al., 2020; Krimigis et al., 1981; Mauk et al., 1996, 2004; Wilson, 2024). They are then used as inputs to study the equilibrium of the magnetodisk. In this regard, the most surprising result may be that the equilibrium is primarily achieved between outward plasma pressure force (including both the gradient and anisotropy force) and inward magnetic stress, while the centrifugal force, which might be thought to be important at first glance, does not contribute too much in general (Caudal, 1986; Mauk & Krimigis, 1987; Paranicas et al., 1991). In addition to equilibrium, plasma observations from Galileo revealed the potential occurrence of a tearing‐mode instability within the magnetodisk (Kronberg et al., 2007). This instability, which would be repeatedly triggered by the interplay of continuous plasma loading from internal sources and field line stretching due to fast planetary rotation, has been proposed to account for the quasi‐periodic burst events observed by Galileo (Krupp et al., 2004). Beyond the magnetodisk itself, magnetodisk plasma observations are also involved in modeling magnetodisk‐ionosphere‐thermosphere coupling (e.g., Cowley & Bunce, 2001; Devinat et al., 2024; Nichols, 2011) and magnetodisk‐moon interaction (e.g., Nordheim et al., 2018, 2022).

Despite these fundamental breakthroughs, uncertainties remain due to the limited temporal and spatial coverage of data. Pioneer, Voyager, Ulysses and New Horizons only flew by Jupiter and thus cannot provide spatial distributions. Although Galileo orbited Jupiter for many years, some instrumental and communication issues restricted primary plasma measurements to regions near the Galilean moons, leaving the magnetodisk outside ∼30 $R_{J}$ not well surveyed (Bagenal et al., 2016) (but offering a full coverage of local times inside this radial distance domain). The arrival of the Juno spacecraft (Bagenal et al., 2017) in July of 2016 offers a new opportunity for studying the magnetodisk plasma. In addition to state‐of‐the‐art charged particle instruments onboard, Juno's orbits are favorable for these studies: They follow a series of polar orbits precessing westward (opposite to Jupiter's rotation) very slowly (as compared to Galileo). This geometry enables one to approximately sample the same local time during a single orbit and to revisit a given position in the radial distance‐local time space a few times within a timescale of several months. These features make it possible to average out temporal variations and obtain fine local‐time resolution when performing statistics. On the other hand, Juno's polar orbit and slow precession limits its local time coverage to a restricted sector, from 18 to 00 and then to 06 hr LT, thus making it impossible to describe the full disk. This limitation will be kept in mind when discussing the impact of our results.

Juno's observations of the magnetodisk, including charged particles (e.g., Kim et al., 2020; Liu et al., 2021; J.‐Z. Wang et al., 2024; Wilson, 2024) and magnetic field (e.g., Connerney et al., 2020; Liu et al., 2021, 2023), have already revealed the broad properties and dynamics of the magnetodisk. Here, we contribute further by generating statistical models of plasma parameters in the equatorial plane and discussing the equilibrium and dynamics of the magnetodisk based on them. To this end, we combine data from the two charged particle instruments onboard Juno: the Jovian Auroral Distributions Experiment (JADE) (McComas et al., 2017) for low‐energy (∼<50 keV) particles and the Jupiter Energetic Particle Detector Instrument (JEDI) (Mauk et al., 2017) for high‐energy (∼>50 keV) particles. This combination is necessary as particles of ∼50 keV dominate energy density, and thus, we cannot accurately measure pressure without either instrument (though this is not a significant issue for number density, to which particles within the JADE energy range contribute dominantly). Besides the two charged particle instruments, magnetic field data obtained by the Juno Magnetic Field investigation (MAG) (Connerney et al., 2017) is also used. This paper is organized as follows. The next section describes the data set and methods. Following it, Section 3 presents plasma density, pressure, temperature and pressure anisotropy and compares our results with the literature. Then, we discuss radial force balance, radial mass transport and particle dynamics based on our observations in Section 4. Finally, Section 5 briefly summarizes our results.

## 2 Methods and Materials

### 2.1 Magnetodisk Crossings

**Figure 2:**
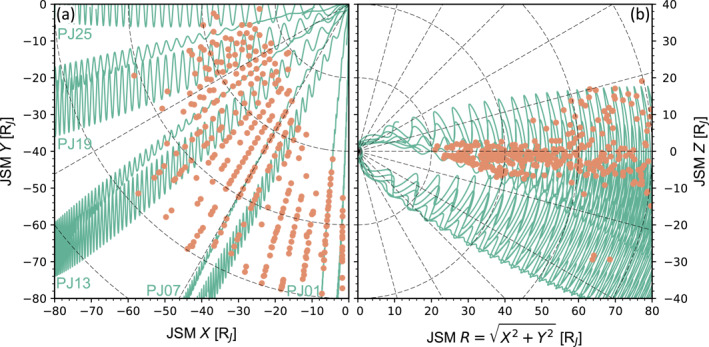
Distributions of the 332 Juno magnetodisk crossings (orange dots) in the (a) equatorial plane and (b) meridian plane of the JSM coordinates. The cyan curves represent Juno's orbits, with orbit numbers denoted in panel (a).

Like all studies on this topic, we begin with the working definition of the magnetodisk. Previous studies usually either simply define the magnetodisk as a specific region in a coordinate system (e.g., Kim et al., 2020), or first identify individual magnetodisk crossings by spacecraft and then extract data near the center of the crossings (e.g., J.‐Z. Wang et al., 2024; Wilson, 2024). Our study adopts the second definition, as it ensures, without ambiguity, that all observations come from the magnetodisk. Here, we use a database of magnetodisk crossings established by Liu et al. (2021) based on magnetic field reversal signatures. These crossings come from the first 25 orbits of Juno (July 2016–July 2020). At large radial distances, the change in the magnetic field associated with magnetodisk crossings, which decreases with increasing radial distance, becomes comparable to the amplitude of temporal fluctuations at larger distances. This results in difficulties in accurately determining the center of the magnetodisk as well as in calculating statistically significant average values of our retrieved parameters. Hence, to ensure the quality of statistics, we only consider crossings that are less than 80 $R_{J}$ from the *z*‐axis of the Jupiter Solar Magnetic (JSM) coordinate system (where the *x*‐axis points from Jupiter to the Sun, the *y*‐axis is perpendicular to both the *x*‐axis and the dipole axis and points to the duskside, and the *z*‐axis completes the right‐handed coordinates). The data set contains 332 crossings. Their distributions in the JSM coordinates are presented as orange dots in Figure 2. This figure also illustrates Juno's orbits PJ01, 07, 13, 19, and 25. It is noted that some crossings could be missing either JADE or JEDI data. Crossings without JEDI data are included when studying number density, while crossings where JADE data are absent are fully excluded from the statistics as none of the plasma moments can be accurately determined.

Figure 2a reveals a strong dependence of radial position on local time. However, this dependence is non‐physical and is caused by the precession of Juno's orbit with time: as time elapses, the ascending node of Juno's orbit gradually approaches Jupiter, which, combined with the westward precession, results in Juno only crossing the magnetodisk at larger and smaller radial distances at later (6 hr) and earlier (0 hr) local times, respectively. This fact also imposes some restrictions on our discussions. Particularly, considerations of radial distributions are limited to intermediate local times (2–5 hr); at earlier or later local times, the outer or inner magnetodisk is not covered. This fact also indicates that most potential magnetodisk crossings by Juno outside 20 $R_{J}$ are included in our case list, although only data before July 2020 is investigated. After this time, Juno crosses the region of interest infrequently.

### 2.2 Data Analysis Framework

The next step is to determine a coordinate system for data presentation. The optimal choice would be a reference frame where the magnetodisk is completely stationary. However, such a coordinate system, even if it exists, is challenging to handle in practice due to the curved geometry (Khurana & Kivelson, 1989) and wavelike motion (Kivelson et al., 1978) of the magnetodisk. Therefore, as a compromise, we seek a coordinate system where the magnetodisk is at rest in a statistical sense.

A possible choice is JSM. As shown in Figure 2b, the identified magnetodisk crossings are concentrated around the equatorial plane, that is, the x‐y plane, of JSM. Although scatter is present, we do not observe any systematic dependence of the z‐coordinate on radial distance (defined as the distance to the *z*‐axis, R) or local time (ϕ) (The ϕ‐dependence of R observed in Figure 2a results from Juno's orbital procession toward the southern hemisphere and is not physical, as mentioned above). We have also checked the statistical distributions of the magnetic field in JSM. The results, presented in Section 4.1, show the statistically averaged magnetic field only reverses direction near the JSM equatorial plane. All these observations indicate the magnetodisk is statistically located near the JSM equatorial plane. Therefore, we present our statistical results in JSM in what follows. More specifically, we will consider two types of distributions: (a) equatorial (x‐y) plane distributions (i.e., radial distance‐local time distributions) and (b) radial profiles (i.e., variables as a function of radial distance) within the 2–5 hr local time sector.

In addition to presenting data, JSM is utilized in discussions of the equilibrium and dynamics of the magnetodisk. For instance, the radial gradient, referenced several times below, is defined as $\frac{\partial }{\partial R}$. Strictly speaking, as formulated in this manner, our results should be viewed as the properties of a statistical magnetodisk, which is a planar disc at the JSM equatorial plane, rather than the statistical properties of the actual magnetodisk, which naturally refers to the average parameters at points moving with the magnetodisk. This distinction becomes evident when, for example, examining the force balance of the magnetodisk (Section 4.1). Due to the wavy nature of the actual magnetodisk, radial force balance, which is discussed here, is generally not equivalent to force balance along the tangential direction of the magnetodisk, which we are really interested in. Overcoming this shortcoming would require that we fit the data to a complex magnetodisk geometry model and do calculations in curved space, which cannot be easily tackled given our limited observations. Hence, we choose the current treatment. We believe our results can be considered, at the very least, as representing a statistical magnetodisk. Although it may deviate somewhat from the actual magnetodisk at a specific time, we believe it captures the fundamental average properties of the latter.

### 2.3 Charged Particle Data

We now move on to the central task of this study—plasma observations. JADE data (JAD‐5‐CALIBRATED‐V1.0/ELC_ANY_DEF and ION_ANY_DEF (Allegrini, Wilson, et al., 2022)) and JEDI data (JED‐3‐CDR‐V1.0/TOF×E (Mauk, 2022)) from NASA's Planetary Plasma Interactions (PPI) node of the Planetary Data System (PDS) are used. We focus on three particle species.

1. Electron (subscript “e”): JADE electron channel plus JEDI electron channel. The first two energy channels (<30 keV) of JEDI are excluded from further analysis due to their anomalous behavior. There is an overlap in energy between JADE and JEDI. Fluxes from the two instruments match well in that range. Therefore, we construct electron velocity distribution functions (VDFs) simply by using JEDI data within its energy range (except for the first two energy channels) and JADE data in the remaining energy range.
2. Proton (subscript “p”): JADE 1 amu/q channel (i.e. SPECIES 3) plus JEDI proton channel. There is also an energy overlap within which the two instruments match each other well. We construct proton VDFs by using JEDI data within its energy range and JADE data in the remaining energy range.
3. Heavy ion (subscript “h”): JADE >5 amu/q channel (i.e., SPECIES 5) plus JEDI oxygen, sulfur and oxygen+surful channels. We assume these ions consist of singly charged oxygen ($O^{+}$) and doubly charged sulfur ($S^{++}$) of equal number density, giving a mean mass of 24 amu and a mean charge of 1.5 e. It is worth noting that previous studies have unveiled the presence of other species within this mass‐charge ratio range, including $S^{+}$, $S^{+++}$ and $O^{++}$ (e.g., J.‐Z. Wang et al., 2024; Wilson, 2024). Thus, our assumption oversimplifies the actual composition. However, calculations using more accurate mean masses and charges (e.g., the ones derived from Figure 10 of J.‐Z. Wang et al. (2024)) do not change the final results much, as the more accurate values are still close to the ones used here, and number density and pressure are only proportional to the square root of mass and charge (see equations below). Therefore, our assumption does not introduce significant errors. Unlike electrons and protons, there is a large energy gap between JADE and JEDI for heavy ions. However, analysis reveals that, in general, fluxes from energy channels near this gap can be fitted to a single power law, indicating the consistency of data from the two instruments. Therefore, we construct heavy ion VDFs by bridging JADE and JEDI with an additional energy channel at $\sqrt{W_{\mathrm{JADE,L}}\times W_{\mathrm{JEDI,F}}}$, where $W_{\mathrm{JADE,L}}$ and $W_{\mathrm{JEDI,F}}$ represent the energy of JADE last channel and JEDI first channel, respectively. Flux at this energy is then determined from a fit of the last three JADE channels and the first three JEDI channels to a power law. This additional energy channel is included when calculating heavy ion moments to account for the contribution from particles within the energy gap between JADE and JEDI.

**Figure 3:**
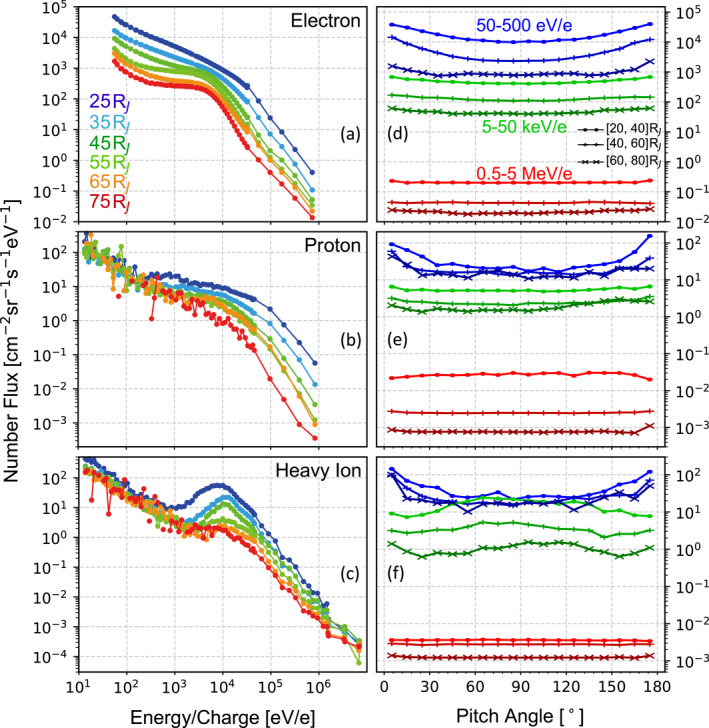
Number flux of charged particles at the center of the magnetodisk, in the rest frame of the spacecraft, within the 2–5 hr local time sector. (a–c) Energy spectra of electrons, protons, and heavy ions at 80°–100° pitch angles. The colors are coded by radial distances (denoted in panel a). For each spectrum, the lower‐energy part with higher resolution is taken from JADE, while the higher‐energy part with lower resolution is taken from JEDI. (d–f) Pitch angle distributions of electrons, protons, and heavy ions. In each panel, the blue, green, and red curves represent spectra at 50–500 eV/e, 5–50 keV/e, and 0.5–5 MeV/e, respectively. The curves marked by “⋅”, “+”, and “×” represent results averaged over 20–40, 40–60, and 60–80 RJ, respectively.

Figure 3 shows the statistical VDFs of the three species in the rest frame of spacecraft. To construct these VDFs, we first extract flux in each crossing by averaging over 5 min centered on the crossing time. Then, we calculate case‐averaged flux in each radial distance bin as detailed in the figure. Figure 3a–3c show energy spectra at 90° pitch angle, with colors indicating radial distances. We only briefly comment on two observations here, leaving a more detailed analysis for the future. First, JADE data (the lower‐energy part with higher energy resolution) and JEDI data (the higher‐energy part with lower energy resolution) connect smoothly without notable jumps. This observation confirms the good cross‐calibration of JADE and JEDI. Second, the energy spectra deviate from Maxwellian‐like distributions and approach power laws at the high‐energy tail, suggesting the influence of kinetic effects on shaping these VDFs.

Figures 3d–3f show the pitch angle distributions at different energy (colors) and radial distance (symbols). Distributions at 50–500 eV/e and 5–50 keV/e are characterized by larger fluxes in the field‐aligned directions, except for the 5–50 keV/e heavy ions, whose distributions peak at 90° pitch angle. These peaks result from the bulk motion of heavy ions (of which peaks near $10^{4}$ eV/e in Figure 3c are another manifestation).

In this study, we do not consider ions with the mass‐charge ratio between 2 and 5 amu/q because their composition (${\text{He}}^{+}$, ${\text{He}}^{++}$, $H_{3}^{+++}$, etc.) is not very clear at present and protons may contaminate their measurements. Fortunately, these ions are minor. A calculation assuming all of them are ${\text{He}}^{+}$ shows that their mass density is an order of magnitude lower than that of heavy ions, and their pressure is two orders of magnitude lower than that of heavy ions. Hence, their presence does not significantly affect our discussions in Section 4.

### 2.4 Numerical Moments

We evenly divide the covered equatorial plane, $R⊗\phi =\left[20,80\right]R_{J}⊗\left[0,6\right]$ hr, into 6×6 bins. Then, we construct statistical VDFs of the three species in each bin using the method described above, obtaining $J_{i}\left(W,\alpha ;R,\phi \right)$ with i=e,h,p, where $J_{i}$ represents differential number flux, and W and α denote particles' energy and pitch angle, respectively. Standard statistical uncertainties $\left(\delta J_{i}\right)$ are also calculated. From the resulting $J_{i}$, we compute number density and pressure using a numerical integration method:

(1) $$N_{i}\left(R,\phi \right)=\sqrt{2\pi^{2}m_{i}}\int W^{-1/2}dW\int \sin\;\alpha d\alpha \cdot J_{i}\left(W,\alpha ;R,\phi \right),$$

(2) $$P_{⊥i}\left(R,\phi \right)=\sqrt{2\pi^{2}m_{i}}\int W^{-1/2}dW\int \sin\;\alpha d\alpha \cdot J_{i}\left(W,\alpha ;R,\phi \right)W\sin^{2}\;\alpha -\frac{1}{2}N_{i}m_{i}V_{⊥i}^{2},$$

(3) $$P_{\|i}\left(R,\phi \right)=\sqrt{8\pi^{2}m_{i}}\int W^{-1/2}dW\int \sin\;\alpha d\alpha \cdot J_{i}\left(W,\alpha ;R,\phi \right)W\cos^{2}\;\alpha -N_{i}m_{i}V_{\|i}^{2},$$

where $V_{i,⊥}$ and $V_{i,\|}$ denote the perpendicular and parallel bulk velocity of i‐th species defined below, and $m_{i}$ represents mass. We also define the anisotropy of the i‐th particle species as:

(4) $$A_{i}\left(R,\phi \right)=P_{\|i}\left(R,\phi \right)/P_{⊥i}\left(R,\phi \right)-1.$$

This definition is used here to maintain consistency with the literature, for example, Mauk and Krimigis (1987) and Paranicas et al. (1991); however, please note that it is different from the one used in wave and instability traditions. Uncertainties of these parameters are then derived from $\delta J_{i}$ using a standard error propagation analysis applied to Equations (1), (2), (3), (4).

**Figure 4:**
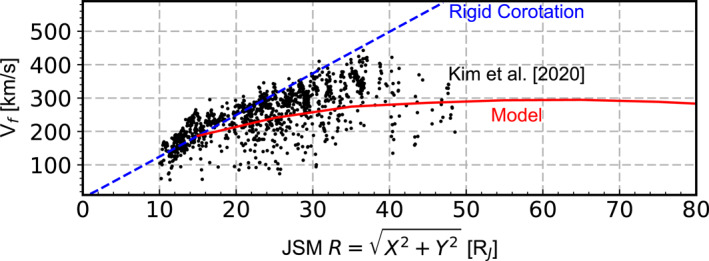
Velocity model used in the calculation of plasma pressure (red curve). The black dots show flow speed obtained by Kim et al. (2020). The blue dashed line represents the rigid corotation speed. This figure is adapted from Figure 6 of Liu et al. (2023).

Velocities are required to calculate pressure. However, a preliminary examination indicates that the velocities derived using the numerical integration method may suffer from large errors, whose sources are currently unclear. Similar issues have also been reported previously, for example, in the processing of the Cassini Plasma Spectrometer data Wilson et al. (2017). On the other hand, published velocity data (e.g., Kim et al., 2020; J.‐Z. Wang et al., 2024; Wilson, 2024) are available only for radial distances less than 50 $R_{J}$, while velocities beyond this range are also required for our calculations. Therefore, here we adopt velocities given by a physical model (Liu et al., 2023) constrained by Juno observations. In a steady state, the electrodynamic torque exerted by the J×B force associated with radial current $\left(I_{r}\right)$ should balance the net transport of angular momentum carried by plasma radial motions. This, by assuming local time symmetry, can be formulated as (Liu et al., 2023; Ray et al., 2014)

(5) $$\dot{M}\frac{d}{dR}\left(Rv_{f}\right)=2\pi R^{2}I_{r}B_{n},$$

where $v_{f}$ is the flow velocity, $\dot{M}$ denotes the radial mass transport rate (in a steady state, it is also the mass loading rate at the inner boundary of the magnetodisk) and $B_{n}$ represents the magnetic field component normal to the magnetodisk. This yields

(6) $$v_{f}\left(R\right)=\frac{R_{0}^{2}}{R}\Omega_{J}+\frac{1}{R}\frac{2\pi }{\dot{M}}\int_{R_{0}}^{R}R^{2}I_{r}B_{n}dR,$$

where $R_{0}$ stands for the “corotation breakdown radius,” that is, a reference radial distance beyond which the magnetodisk starts departing significantly from rigid corotation with the planet. Figure 4, which is adapted from Figure 6 of Liu et al. (2023), shows $v_{f}$ given by this equation (red curve), using $I_{r}$ and $B_{n}$ measured by Juno (Liu et al., 2023), $\dot{M}=$1500 kg/s and $R_{0}=$15 $R_{J}$. The results fit the flow speed obtained by Kim et al. (2020) (black dots) well. The validity of this velocity model is further supported by the fact that the kinetic energy it predicts is comparable to the peak energy observed in the energy spectra of heavy ions (Figure 3c). Therefore, $v_{f}$ obtained in this way is used as $V_{⊥i}$ in Equation 2. We further set $V_{\|i}$ in Equation 3 to zero, since it represents the velocity component normal to the magnetodisk and is approximately zero, as demonstrated by J.‐Z. Wang et al. (2024) and Wilson (2024).

## 3 Observations

**Figure 5:**
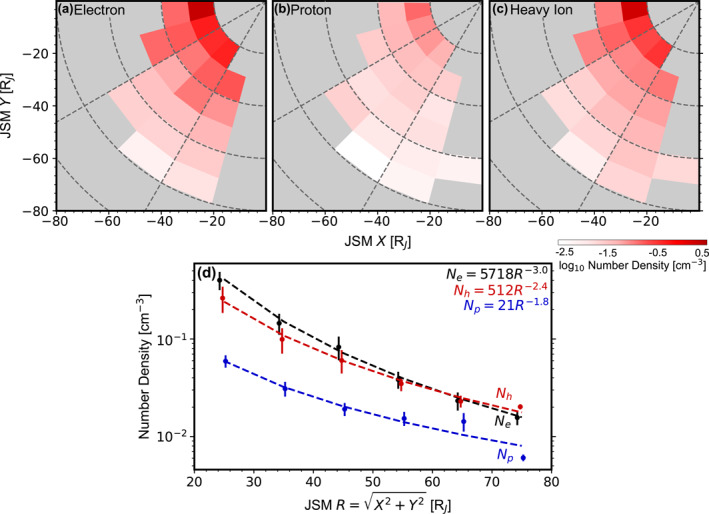
Number density. (a–c) Distributions in the JSM equatorial plane of the number density of electrons, protons and heavy ions. Regions not covered by Juno are shown in gray. (d) Radial profiles of the number density of electrons (black), protons (blue) and heavy ions (red) within the 2–5 hr local time sector. The dashed curves show the fit of power laws to the observations, with the results denoted in this panel.

**Figure 6:**
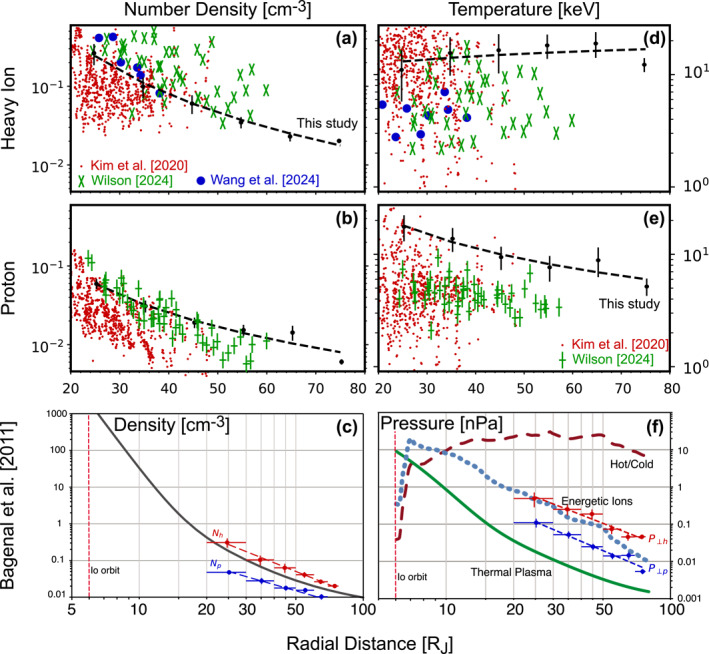
Comparison of the results obtained in this study with those in previous publications. (a) Number density of heavy ions obtained by Juno: this study (black dots and curve), Kim et al. (2020) (red dots), Wilson (2024) (green crosses), and J.‐Z. Wang et al. (2024) (blue solid circles). (b) Number density of protons obtained by Juno: this study (black dots and curve), Kim et al. (2020) (red dots), and Wilson (2024) (green crosses). (c) Number density obtained by our study (red and blue dots connected by dashed curves) and by Bagenal and Delamere (2011) from missions before Juno (black solid curve). (d and e) Temperature obtained by Juno, with the same format as panels (a and b). (f) Pressure obtained by our study (red and blue dots connected by dashed curves) and by Bagenal and Delamere (2011) from missions before Juno (green solid, blue dotted, and red dashed curves).

**Figure 7:**
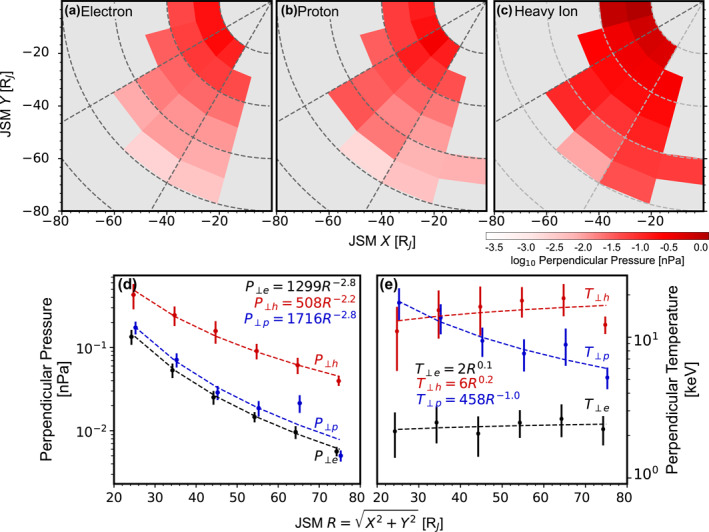
(a–d) Perpendicular pressure, with the same format as Figure 5. (e) Perpendicular temperature of electrons (black), protons (blue) and heavy ions (red).

**Figure 8:**
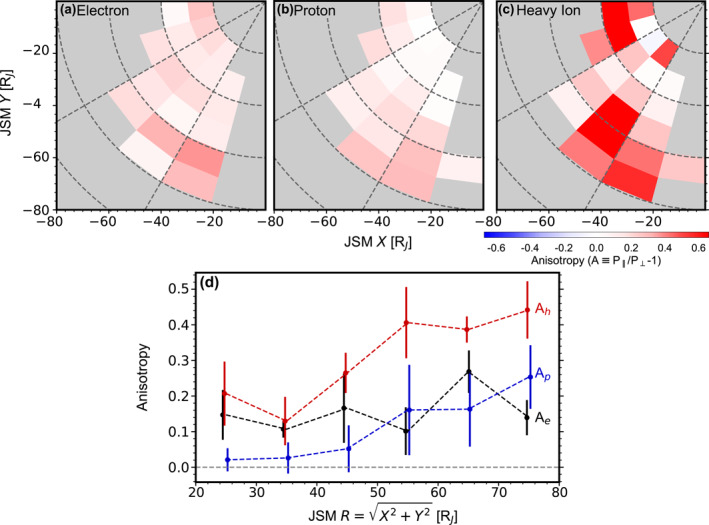
Pressure anisotropy $\left(P_{\|}/P_{⊥}-1\right)$, with the same format as Figure 5.

This section presents the statistical distributions of the plasma parameters defined above. Errors are presented as error bars when plotting radial profiles. Besides, our results are compared with previous studies. Results presented in this section are summarized in Table 1. The reader familiar with the context who wants to save time may quickly go through the text and then examine this table as well as Figures 5, 6, 7, 8 by themselves.

**Table 1:** Plasma Moments Within 2–5 Local Time

		Radial distance, R ${[R}_{J}$]			
		20	50	80	Power‐law fit
N ${[\text{cm}}^{-3}$]	e	0.273	0.061	0.019	$5718R^{-3.0}$
	h	0.181	0.048	0.021	$512R^{-2.4}$
	p	0.045	0.017	0.010	$21R^{-1.8}$
$P_{⊥}$ [nPa]	e	0.093	0.020	0.008	$1299R^{-2.8}$
	h	0.337	0.124	0.051	$508R^{-2.2}$
	p	0.122	0.023	0.013	$1716R^{-2.8}$
$T_{⊥}$ [keV]	e	2.31	2.27	2.43	$2R^{0.1}$
	h	13.28	17.33	15.58	$6R^{0.2}$
	p	15.67	8.54	7.00	$458R^{-1.0}$
$A=\frac{P_{\|}}{P_{⊥}}-1$	e	0.13	0.14	0.20	–
	h	0.17	0.34	0.41	–
	p	0.02	0.11	0.21	–

### 3.1 Number Density

Figures 5a–5c show the equatorial plane distributions of the number density of electrons, protons and heavy ions, respectively. No dependence on local time is identified within the covered range of local times. However, a clear decreasing trend with increasing radial distances can be observed for all species. This point is illustrated more clearly in Figure 5d, which presents the radial profiles averaged over the 2–5 hr local times. Additionally, as shown by the dashed curves, these profiles can be fitted to power laws:

(7) $$N_{e}=\left(5.7\pm 2.1\right)10^{3}R^{-\left(3.0\pm 0.1\right)},N_{p}=\left(2.1\pm 2.1\right)10^{1}R^{-\left(1.8\pm 0.3\right)},N_{h}=\left(5.1\pm 2.4\right)10^{2}R^{-\left(2.4\pm 0.1\right)},$$

where R is in $R_{J}$ and $N_{i}$ is in ${\text{cm}}^{-3}$. The resulting exponents vary among species, which may indicate differences in the sources, sinks, or transport history of different species. The radial profiles also show that $N_{h}$ is twice or third of $N_{p}$. Thus, heavy ions are the majority species among ions. Furthermore, a calculation indicates the ratio $\left(1.5N_{h}+N_{p}\right)/N_{e}$ (where the factor 1.5 is the mean charge of heavy ions) varies from 1.2 to 2.3 over the considered radial extent (no systematic dependence of this ratio on radial distances is noted). Since this ratio is not far from unity as required by the quasi‐neutrality condition, we suggest the bulk of the magnetodisk plasma must have been captured by JADE+JEDI in the data we are using.

Figures 6a and 6b compare our number density with that derived from a forward modeling analysis of JADE data. Regarding heavy ions, our statistical values are situated at the upper end of the range reported by Kim et al. (2020), at the lower end of Wilson (2024)'s results, and close to those by J.‐Z. Wang et al. (2024). For protons, our statistical values are aligned with the upper range observed by Kim et al. (2020) and closely resemble the results by Wilson (2024). Differences emerge from these comparisons. Nevertheless, the results obtained by different studies are still comparable in terms of both absolute values and radial trends. Figure 6c further compares our results with the model generated by Bagenal and Delamere (2011) based on missions before Juno. Our $N_{h}$ and $N_{p}$ are of the same order of magnitude as the model results, indicating there has not been any significant change in plasma number density between different missions.

### 3.2 Perpendicular Pressure and Temperature

Figures 7a–7d show the perpendicular pressure. No clear dependence on local time is observed for electrons and heavy ions, while proton pressure exhibits an increasing trend toward midnight at a fixed radial distance. On the other hand, the perpendicular pressure for all species shows a clear decreasing trend with increasing radial distance. Figure 7d presents the radial profiles averaged over the 2–5 hr local times. The profiles can be fitted to power laws:

(8) $$P_{⊥e}=\left(1.3\pm 0.3\right)10^{3}R^{-\left(2.8\pm 0.1\right)},P_{⊥p}=\left(1.7\pm 1.5\right)10^{3}R^{-\left(2.8\pm 0.5\right)},P_{⊥h}=\left(5.1\pm 2.8\right)10^{2}R^{-\left(2.2\pm 0.2\right)},$$

where R is in $R_{J}$ and $P_{⊥i}$ in nPa. In addition, we note $P_{⊥h}$ is the largest everywhere within the covered regions, being 2–5 times that of $P_{⊥p}$, which is close to, but slightly larger than, $P_{⊥e}$.

From the number density and perpendicular pressure, we also derive an effective perpendicular temperature as $T_{⊥i}=P_{⊥i}/N_{i}$ (in energy unit). Figure 7e presents the results. $T_{⊥e}$ and $T_{⊥h}$ only change slightly over the covered radial distance, respectively increasing from 2.31 and 13.28 keV at 20 $R_{J}$ to 2.43 and 15.58 keV at 80 $R_{J}$. On the other hand, $T_{⊥p}$ shows a notable decrease, from 15.67 keV at 20 $R_{J}$ to 7.00 keV at 80 $R_{J}$.

Figures 6d and 6e compare our perpendicular temperature with previous publications. Strictly speaking, the previous results cited here were obtained under an assumption of isotropic VDFs. Thus, we might consider introducing an effective isotropic temperature $\left(T_{\mathrm{iso}}\right)$ before comparing it with these earlier results. However, accordingly to the anisotropy presented in the next subsection, $T_{⊥}$ should not differ significantly from $T_{\mathrm{iso}}$. For instance, if we define $T_{\mathrm{iso}}$ as $\left(T_{\|}+2T_{⊥}\right)/3$, one will find that $T_{\mathrm{iso}}/T_{⊥}$ ranges from ∼1.06 at 20 $R_{J}$ to ∼1.13 at 80 $R_{J}$ for heavy ions (this ratio is even closer to unity for electrons and protons). Therefore, we neglect the distinction between $T_{⊥}$ and $T_{\mathrm{iso}}$ in comparison for the sake of simplicity. For heavy ions, our statistical values are close to median values obtained by Kim et al. (2020) but larger than those by Wilson (2024) and J.‐Z. Wang et al. (2024). For protons, our statistical values are larger than the results obtained by Kim et al. (2020) and Wilson (2024). However, we note that all results shown here are of the same order of magnitude. Figure 6f compares our perpendicular pressure with the model generated by Bagenal and Delamere (2011). Our $P_{⊥h}$ is fairly close to the energetic ion pressure given by the model, indicating the absence of significant changes in pressure between different missions.

We do not present parallel pressure here to save space. Nevertheless, one can easily recover it from the perpendicular pressure shown here and the anisotropy in the following subsection.

### 3.3 Anisotropy

Figure 8 presents anisotropy. It is positive on average for all species, indicating their VDFs are dominated by parallel motion rather than perpendicular motion, a point we have already noted from Figures 3d–3f. However, upon examining the time series of individual crossings, we observe that anisotropy can occasionally become negative during certain periods. Future studies may address the specific conditions that lead to different signs of anisotropy.

The anisotropy distributions fluctuate more than those of density and pressure. Nevertheless, a clear increasing trend with radial distances is noted for all species. For example, Figure 8d shows the radial profiles of anisotropy averaged over the 2–5 hr local time sector. From 20 to 80 $R_{J}$, $A_{h}$ grows from 0.17 to 0.41, $A_{p}$ from 0.02 to 0.21, and $A_{e}$ from 0.13 to 0.20. On the other hand, we do not observe any systematic trend regarding local time in Figures 8a–8c.

Previous reports on anisotropy are rare. One frequently cited study is by Paranicas et al. (1991), who showed the anisotropy of oxygen and protons are about 0.2 at 20–30 $R_{J}$ as observed by Voyager. These results are consistent with ours.

## 4 Discussion

In this section, we discuss some properties of the magnetodisk based on the observations presented above. Before proceeding, we would like to point out that our data were obtained during solar minimum, when solar wind conditions are generally expected to be quiet. During solar maximum, the interaction between the solar wind and the magnetosphere is expected to be stronger, which may alter some of these properties. Future studies comparing observations from Juno with those from other missions, as well as future joint studies of Juno's prime and extended mission data to broaden local time coverage, may help to address these effects.

### 4.1 Radial Force Balance

Our observations provide clues for the equilibrium and dynamics of the magnetodisk. We first examine the radial force balance of the magnetodisk, which can be written as

(9) $$-\frac{\partial }{\partial R}\frac{B^{2}}{2\mu_{0}}+\rho \frac{v_{f}^{2}}{R}-\frac{\partial }{\partial R}P_{⊥}-AP_{⊥}\kappa_{r}+\frac{B^{2}}{\mu_{0}}\kappa_{r}=0,$$

where $\rho =\sum m_{i}N_{i}$, $P_{⊥}=\sum P_{⊥i}$, $A=\sum P_{\|i}/\sum P_{⊥i}-1$ represent the total mass density, perpendicular pressure and anisotropy of plasma, respectively, B and $\kappa_{r}$ are magnetic field strength and radial curvature of magnetic field lines at the center of the magnetodisk, and $\mu_{0}$ represents permeability of vacuum. Each term of this equation represents a particular force. From the left to the right, they are magnetic pressure force, centrifugal force, pressure gradient force, anisotropy force and magnetic stress force.

To evaluate this equation, we first need to evaluate the two parameters associated with magnetic field, B and $\kappa_{r}$. B can be obtained easily. The calculation of $\kappa_{r}$ is more complicated. In principle, it requires the knowledge of three‐dimensional magnetic field. Fortunately, what follows will show that the radial force balance is primarily achieved between the anisotropy force and magnetic stress force. Since they are both proportional to $\kappa_{r}$, uncertainty in $\kappa_{r}$ would not affect our overall conclusion much. Previous studies usually derive $\kappa_{r}$ by fitting the observed magnetic field near individual magnetodisk crossings to some prescribed models, and then determine $\kappa_{r}$ from the fit. Given the much larger database used here, we derive $\kappa_{r}$ differently. We first generate a statistical model of the magnetic field and then calculate $\kappa_{r}$ (as well as B) from the distributions. We wish to point out that, as mentioned in Section 2.2, the magnetodisk we analyzed here is a “statistical” representation and not a snapshot of any specific time. The averaging process may partially smooth out vertical variations in the magnetic field, potentially leading to an underestimate of $\kappa_{r}$. Fortunately, this does not impact our analysis much, as we will demonstrate below that radial force balance is primarily governed by magnetic stress force and anisotropy force, which would be even larger than calculated if this effect were real.

**Figure 9:**
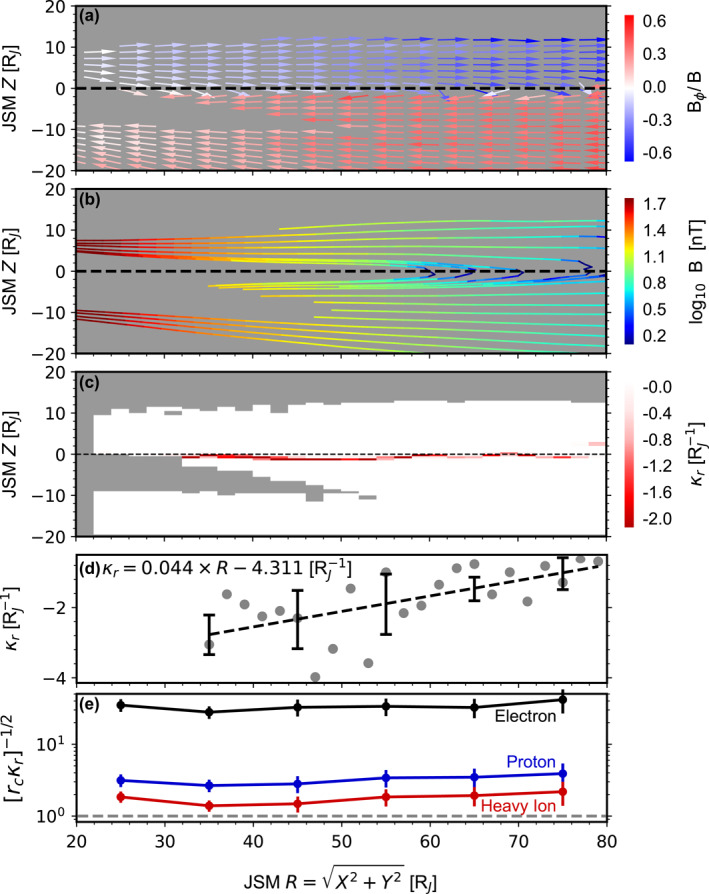
Statistics of the magnetic field within the 2–5 hr local time sector. (a) Projection of the magnetic field vectors on the JSM meridian plane. The colors represent the ratio of the azimuthal component to the total magnetic field strength. Reds and blues correspond to eastward and westward directions, respectively. (b) Projection of the magnetic field lines on the JSM meridian plane, derived from the vector field shown in panel (a). The colors represent the magnitude of the magnetic field. (c) Radial component of curvature $\left(\kappa_{r}\right)$ derived from the field lines shown in panel (b). A minus sign indicates the radial inward direction. (d) Radial profile of the maximum $\kappa_{r}$ at each radial distance. The black dashed line shows a fit to a linear function. (e) Square root of the ratio of radial curvature radius $\left(1/\kappa_{r}\right)$ to particle gyro‐radius $\left(r_{c}\right)$, which is evaluated at the perpendicular temperature presented in Figure 7e. The black, red and blue curves represent electrons, heavy ions and protons, respectively.

To generate this statistical model of the magnetic field, we first evenly divide the sampled region into many bins and calculate the mean magnetic field measured by Juno in each bin. This process creates a look‐up table that provides the magnetic field at discrete points, that is, the center of these bins. Figure 9a shows the results within the 2–5 hr local time sector (the resolution of bins has been reduced for the sake of presentation). The vectors represent the projection in the meridian (R‐z) plane, whereas the colors give the ratio of the off‐meridian (i.e., azimuthal) component $\left(B_{\phi }\right)$ to the magnetic field strength. One may note that the magnetic field maintains the same direction above or below the equatorial plane, and reverses direction near the equatorial plane, indicating that the magnetodisk is located near the JSM equatorial in a statistical sense. Additionally, $B_{\phi }$ is negative (i.e., pointing westward) above the equatorial plane and positive (i.e., pointing eastward) below it, suggesting a well‐known sweepback configuration. To calculate $\kappa_{r}$, we then trace field lines by solving the field line equation, using the magnetic field at any arbitrary position obtained through linear interpolation from the look‐up table of the statistical magnetic field. A fourth‐order Runge‐Kutta method is employed for the numerical computation. Figure 9b shows the resulting field lines. The colors represent the magnetic field strength, from which B is obtained. $\kappa_{r}$ is then determined from the field lines as the radial component of the curvature vector $\frac{d^{2}\vec{\zeta }}{ds^{2}}$, where $\vec{\zeta }$ is a vector pointing from an arbitrary origin (taken as the JSM origin here) to positions along a single field line and s is distance along the field line. Figure 9c shows the distributions of the resulting $\kappa_{r}$ (regions without valid data are marked by gray color). The negative sign indicates the curvature vectors point toward smaller radial distances. As expected, the magnitude of $\kappa_{r}$ peaks near the equatorial plane and is nearly zero at other positions. Figure 9d shows the minimum $\kappa_{r}$ (i.e., maximum magnitude) at each radial distance. Despite some scatter, one may observe that the magnitude of $\kappa_{r}$ decreases as radial distance increases. These data points are further fitted to a linear function (the dashed line). This fit yields curvature radii $\left(1/|\kappa_{r}|\right)$ of about 0.28, 0.30 and 0.36 $R_{J}$ at radial distances 18.0, 23.1, and 35.5 $R_{J}$, respectively, which are comparable with those obtained by Paranicas et al. (1991): 0.17, 0.30, 0.55 $R_{J}$. The fitting results are used to evaluate radial force balance.

**Figure 10:**
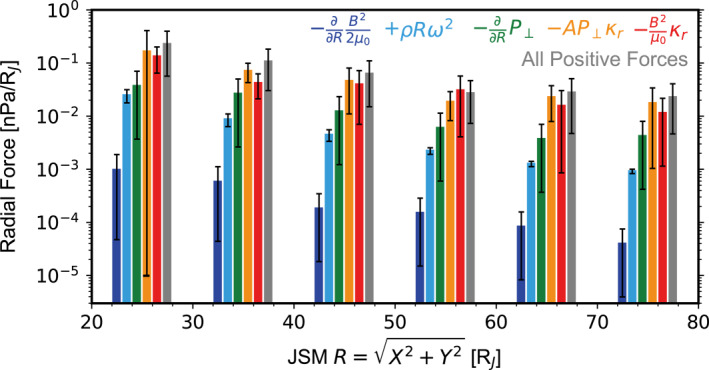
Radial force balance at different radial distances. The blue, light blue, green, orange and red bars represent the magnetic pressure force, centrifugal force, plasma pressure force, plasma anisotropy force and magnetic stress force, respectively. The gray bars represent the summation of all positive forces, that is, all forces except the magnetic stress force.

Figure 10 evaluates the five force terms defined by Equation 9 at different radial distances, utilizing the plasma and magnetic field parameters detailed above (where $v_{f}$ from Equation 6 is employed to calculate the centrifugal force). All forces are positive, that is, directing outward, except for the magnetic stress force (thus, its absolute value is shown). The gray bars represent the summation of the four outward forces. The ratio of this summation to the inward magnetic stress force (red bars) is close to unity, ranging from 0.9±1.0 to 2.5±2.2. We do not observe any systematic dependence of the unbalance on radial distances. Therefore, we attribute the unbalance to uncertainties in data and conclude that our “statistical magnetodisk” is in radial force balance.

Among the four positive forces, the anisotropy force (orange bars) is dominant, accounting for 73% of the total positive forces on average. The pressure gradient force (green bars) is secondary and contributes 19% on average. Finally, contributions from the centrifugal force (light blue bars; 7%) and the magnetic pressure force (dark blue bars; <1%) are not important overall. Our results thus support a long‐lasting viewpoint (Mauk & Krimigis, 1987; Paranicas et al., 1991) that plasma anisotropy plays a dominant role in the equilibrium of the magnetodisk. Historically, the magnetodisk was usually modeled by assuming an isotropic plasma. Our results suggest that these results may deviate from the actual magnetodisk, and future modeling work should take plasma anisotropy as an essential aspect. In fact, Nichols et al. (2015) already noted that the inclusion of plasma anisotropy in the modeling of the magnetodisk could result in better consistency with observations, especially when considering its variability.

### 4.2 Kappa Parameter

Our observations also provide insight into particle kinetics. For instance, Figure 9e presents the so‐called kappa parameter, which is defined as the square root of the ratio of the curvature radius $1/|\kappa_{r}|$ to the gyro‐radius of particles $r_{c}=\frac{\sqrt{2m_{i}T_{⊥i}}}{|q_{i}|B}$ (where $q_{i}$ is the charge). This parameter serves as an index of the stochasticity in particle motion (e.g., Büchner & Zelenyi, 1989; Zelenyi et al., 2013, and reference therein). The kappa parameter of electrons (black) is approximately 30 over the entire covered radial extent. Therefore, solely considering the current sheet dynamics, electrons are expected to be fully adiabatic with magnetic moments conserved. This fact immediately suggests that $T_{⊥e}$ should decrease as radial distances increase (i.e., adiabatic cooling), given that the magnetic field is weaker toward the outer regions. However, the observed $T_{⊥e}$ (Figure 7e) shows a weak increasing trend. This contrast indicates the presence of some heating mechanisms, such as wave‐particle interaction and turbulent dissipation.

The kappa parameter of protons (blue curve in Figure 9e) increases from ∼3 at 20 $R_{J}$ to ∼4 at 80 $R_{J}$. Therefore, over the considered radial extent, they are located near the boundary between adiabatic and stochastic regimes (e.g., region (2) in Figure 19 of Büchner and Zelenyi (1989)) and would slowly diffuse in magnetic moment space. Finally, the kappa parameter of heavy ions (red curve in Figure 9e) ranges from 1.4 to 2.3 over the considered radial extent. Thus, their motion is expected to be fully stochastic, resulting in rapid and irregular transport in velocity space (and possibly in coordinate space as well (Birmingham, 1985)). This stochastic transport provides a heating mechanism and may account for the superthermal tail observed in VDFs. Additionally, it would cause strong pitch angle scattering, leading to isotropic pitch angle distributions and anisotropy close to unity. However, our observations (Figure 8) show that the anisotropy of heavy ions is greater than unity on average. It even increases with radial distance or, equivalently, with time, assuming that plasma is transported radially outward. These facts together suggest that there are mechanisms that continuously increase anisotropy along particle trajectories in action. The strong scattering also indicates heavy ions are not fully frozen in with the field lines. On the other hand, electrons are expected to do so as their motion is adiabatic. The differential motion between heavy ions and electrons may give rise to finite anomalous resistivity in the magnetodisk, enabling the electric field here to accelerate/heat particles and derive currents.

### 4.3 Radial Transport

Radial transport is central in any discussions of the magnetodisk. Our results also provide some clues. Previous studies have demonstrated that the magnetodisk is asymmetric in local time. Hence, discussions on radial transport require, in principle, plasma distributions at all local times. However, as mentioned in Section 2.1, Juno only covers the magnetodisk outside 20 $R_{J}$ in a limited local time sector. For Juno to make any meaningful contribution, we have to assume that our results, derived from its limited observations, can be extrapolated to other local times, which is equivalent to assuming that the magnetodisk is symmetric. Despite the obvious deviation from reality, this assumption allows us to estimate some key parameters.

**Figure 11:**
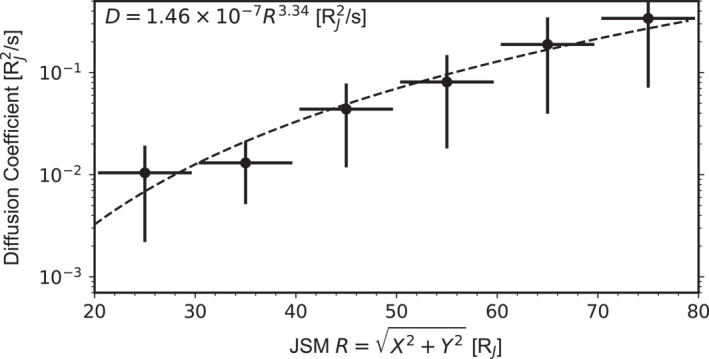
Diffusion coefficient, derived from the statistical radial profiles of density, with the dashed curve representing its fit to a power law.

First of all, aligned with previous studies (e.g., Russell et al., 2000), we estimate the rate of radial transport from our mass density profile, ρ(R). Due to the lack of knowledge of the detailed mechanisms, we simplify the radial transport as a one‐dimensional diffusion process,

(10) $$\frac{\partial \rho }{\partial t}+\frac{1}{R}\frac{\partial }{R}\left(2\pi RD\frac{\partial \rho }{\partial R}\right)=0,$$

where D is diffusion coefficient. By further assuming diffusive equilibrium, we have

(11) $$2\pi RhD\frac{\partial \rho }{\partial R}=\dot{M},$$

or,

(12) $$D\left(R\right)=\frac{\dot{M}}{2\pi Rh\left(\partial \rho \right)/\left(\partial R\right)},$$

where $\dot{M}$ denotes the mass loading rate at the inner boundary (Io orbit) and h represents the thickness of the magnetodisk. Based on the same data set used in this study, Liu et al. (2021) (their Figure 2c) calculated the thickness of the magnetodisk from a Harris fit of these crossings, and Liu et al. (2023) (their Figure 6 and Figure 4 of this paper) showed $\dot{M}∼$1,500 kg/s fit Juno's electric current and flow velocity observations best. Figure 11 shows D obtained with these results. As indicated by the dashed curve, it can be fitted to a power law,

(13) $$D\left(R\right)=\left(1.46\pm 2.42\right)\times 10^{-7}R^{3.34\pm 0.43},$$

with R in $R_{J}$ and D in $R_{J}^{2}$/s. The resulting exponent is positive and significant, suggesting that radial transport is much faster at larger radial distances. In accordance with the definition of D, we estimate the timescale of the radial transport from 20 $R_{J}$ to 80 $R_{J}$: $\tau_{D}=\sum_{i}\frac{{\left(\delta R_{i}\right)}^{2}}{D_{i}}∼$7 hr, where the subscript i runs over the six data points in Figure 11. It is interesting to expand our result to radial distances smaller than 20 $R_{J}$ and estimate a timescale of radial transport there, finding ∼50 hr for plasma flowing from the Io orbit to 20 $R_{J}$. This value is about seven times $\tau_{D}$, although the corresponding radial displacement is only 20% of that of the latter. This estimation intuitively illustrates how slow radial transport is in the inner magnetosphere.

We also estimate the radial transport timescale from another perspective. First, we calculate the total mass of the magnetodisk between 20 and 80 $R_{J}$ by integrating ρ(R) over this radial extent, $M=\int dR\cdot 2\pi Rh\cdot \rho =\left(5.0\pm 0.7\right)\times 10^{7}$ kg. Bagenal and Delamere (2011) derived the distribution of mass of the magnetodisk with missions before Juno (their Figure 7). By calculating the area below their curve, we find that their distribution gives a total mass of $9.6\times 10^{7}$ between 20 and 80 $R_{J}$, comparable to our figure. With the obtained M, we then estimate the timescale for refreshing the magnetodisk plasma, which is also the timescale for radial transport, $\tau_{M}∼\frac{M}{\dot{M}}∼9.2$ hr. This estimate is close to $\tau_{D}$.

We can further comment on the energy budget during radial transport with out distributions. To this end, we calculate the total thermal energy of the magnetodisk between 20 and 80 $R_{J}$: $W_{th}=\int dR\cdot 2\pi Rh\cdot \frac{1}{2}\left(2P_{⊥}+P_{\|}\right)=\left(3.8\pm 0.3\right)\times 10^{37}$ eV. We further estimate the initial total thermal energy with the total mass between 20 and 80 $R_{J}$ and temperature at 20 $R_{J}$: $W_{th,20}=\left(3.3\pm 0.2\right)\times 10^{37}$ eV. The difference is positive and marginally larger than the error: $W_{th}-W_{th,20}=\left(0.5\pm 0.4\right)\times 10^{37}$ eV. This result indicates that the plasma is heated when transported from 20 to 80 $R_{J}$. It also provides a hint for the existence of energy sources outside the magnetodisk plasma since plasma bulk velocity (Figure 4) and thus kinetic energy also increase during the transport.

### 4.4 Application to the JUICE Mission

**Figure 12:**
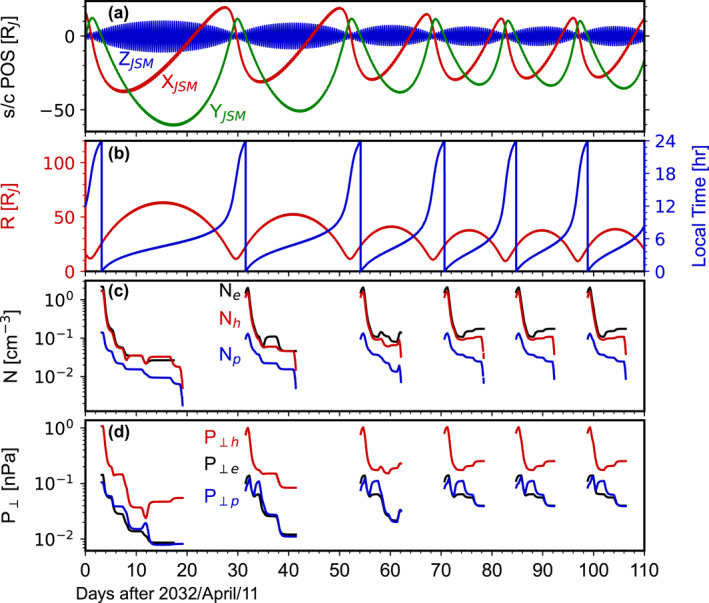
Virtual observations of JUICE during its early phase orbiting Jupiter, synthesized based on the statistical distributions obtained in this study. (a) JUICE orbits in JSM, with red, green and blue curves corresponding to x, y, and z coordinates. (b) Radial distance to the JSM *z*‐axis (red) and local time (blue). (c) Number density of electrons (black), heavy ions (red) and protons (blue). (d) Perpendicular pressure of electrons (black), heavy ions (red) and protons (blue).

The plasma distributions presented in this paper also serve as reference for future missions. For instance, Figure 12 illustrates what the JUpiter ICy moons Explorer (JUICE) mission may observe during its early phase based on our model. Strictly speaking, we cannot predict real‐time values along JUICE's orbit since we do not incorporate any magnetodisk geometry model into our model and thus cannot accurately determine when JUICE will cross the magnetodisk. Similar to Futaana et al. (2018), here we synthesize virtual observations by first identifying JUICE's position in the radial distance‐local time space at a given time and then equating the desired variables to the values given by our model at this position. Consequently, Figure 12 may be considered as describing what JUICE would observe if it were to cross the magnetodisk at this radial distance and local time. Despite this oversimplification, this figure should capture some basic features. For example, it shows that the temporal profile of plasma observations obtained by JUICE during a single orbit may be non‐monotonic due to JUICE's large excursion in local time near its apojove.

## 5 Summary

In this study, we investigated Juno's observations of the magnetodisk plasma within the radial distance range of 20–80 $R_{J}$ and the local time sector of 0–6 hr. By combining data from the JADE and JEDI instruments, we generated spatial distributions of the number density, pressure, and velocity of electrons, protons and heavy ions. Then, based on these distributions, we discussed the equilibrium and dynamics of the magnetodisk. The main results are summarized as follows.

1. Heavy ions are the dominant ion species both in terms of number density and pressure.
2. The variation with radial distance of the number density and pressure of all species can be fitted to a power law of radial distance with negative exponents. As radial distance increases, the effective perpendicular temperature slowly increases for electrons and heavy ions and decreases for protons.
3. On average, the VDFs of all species are anisotropic, displaying the parallel pressure larger than the perpendicular pressure. Anisotropy increases with radial distance for all species.
4. In the 20–80 $R_{J}$ range of this study, the magnetic stress force is the only force pointing inward. Regarding the outward forces, the anisotropy force is dominant (73%), the pressure gradient force is secondary (19%), and the centrifugal force (7%) and magnetic pressure force (<1%) are not important in general. The magnetodisk is approximately in radial force balance.
5. The kappa parameter is about 30 for electrons, 3–5 for protons, and 1–2 for heavy ions, implying that the bulk of the three species undergo adiabatic motion, magnetic moment diffusion and fully stochastic motion, respectively.
6. We derive from the radial mass profile a radial diffusion coefficient by assuming diffusive equilibrium and local time symmetry. The resulting diffusion coefficient can be fitted to a power law with an exponent of 3.34. From it, we estimate that the timescale of radial transport from 20 to 80 $R_{J}$ is on the order of ∼7 hr.
7. We also provide estimates of the total mass ($(5.0\pm 0.7)\times 10^{7}$ kg) and thermal energy ($(3.8\pm 0.3)\times 10^{37}$ eV) of the magnetodisk between 20 and 80 $R_{J}$. The estimate of the total mass leads to another estimate of the radial transport timescale, ∼10 hr.

A comparison of our results with those obtained by previous missions does not reveal significant differences. We hope our results will serve as a useful reference for further missions. As an illustration, we generated what the JUICE mission would observe during the magnetodisk crossings of its early phase based on our findings.

## Data Availability

The authors acknowledge the use of NASA's Planetary Plasma Interactions (PPI) node of the Planetary Data System (PDS) for obtaining the Juno/MAG (PDS volume FGM‐CAL‐V1.0/SS) data (Connerney, 2022), the Juno/JADE (PDS volume JAD‐5‐CALIBRATED‐V1.0/ELC_ANY_DEF and ION_ANY_DEF) data (Allegrini, Wilson, et al., 2022), and the Juno/JEDI (PDS volume JED‐3‐CDR‐V1.0/TOF×E) data (Mauk, 2022).
